# Intersectional Gene Expression in Zebrafish Using the Split KalTA4 System

**DOI:** 10.1089/zeb.2015.1086

**Published:** 2015-12-01

**Authors:** Rafael Gois Almeida, David Anthony Lyons

**Affiliations:** ^1^ Centre for Neuroregeneration, University of Edinburgh, Edinburgh, United Kingdom.; ^2^ MS Society Centre for Translational Research, University of Edinburgh, Edinburgh, United Kingdom.; ^3^ Euan MacDonald Centre for Motor Neurone Disease Research, University of Edinburgh, Edinburgh, United Kingdom.

## Abstract

In this study, we describe the adaptation of the split Gal4 system for zebrafish. The Gal4-UAS system is widely used for expression of genes-of-interest by crossing driver lines expressing the transcription factor Gal4 (under the control of the promoter of interest) with reporter lines where upstream activating sequence (UAS) repeats (recognized by Gal4) drive expression of the genes-of-interest. In the Split Gal4 system, hemi-drivers separately encode the DNA-binding domain (DBD) and the activation domain (AD) of Gal4. When encoded under two different promoters, only those cells in the intersection of the promoters' expression pattern and in which both promoters are active reconstitute a functional Gal4 and activate expression from a UAS-driven transgene. We split the zebrafish-optimized version of Gal4, KalTA4, and generated a hemi-driver encoding the KalTA4 DBD and a hemi-driver encoding KalTA4's AD. We show that split KalTA4 domains can assemble *in vivo* and transactivate a UAS reporter transgene and that each hemi-driver alone cannot transactivate the reporter. Also, transactivation can happen in several cell types, with similar efficiency to intact KalTA4. Finally, in transient mosaic expression assays, we show that when hemi-drivers are preceded by two distinct promoters, they restrict the expression of an UAS-driven reporter from a broader pattern (*sox10*) to its constituent smaller neuronal pattern. The Split KalTA4 system should be useful for expression of genes-of-interest in an intersectional manner, allowing for more refined manipulations of cell populations in zebrafish.

## Introduction

Ectopic expression of genes-of-interest (GOI) in cells is a fundamental method used to understand the function of genes and the biology of cells. Driving expression of a fluorescent protein, for instance, illuminates the target cells' morphology, origin, and behavior.^1^ Expression of optogenetic tools permits interrogation or manipulation of the physiological state of the target cell.^2^ Expressing wild-type or mutant alleles of GOI helps elucidate their role in the specification, development, or function of target cells or tissues.^3^ In a recent effort to systematically generate mutations in each protein-coding gene of the zebrafish genome, it was determined that over 80% of nonsense or essential splice mutations, representing over 1200 genes, have no overt morphological or behavioral phenotype within 5 dpf.^4^ Functional data for many of these genes may arise from studies resorting to ectopic expression of their wild-type and mutant forms.

One manner in which to direct gene expression to target cells is to use upstream regulatory sequence fragments, typically from promoters, known to be expressed in the desired cell type. Such constructs are typically injected into fertilized eggs, resulting in transient, mosaic expression in the target cells. These animals can be raised and their offspring assessed for genome integration of the construct, which results in stable, nonmosaic expression in all target cells in the animal.^5^ A number of approaches greatly increase the efficiency of genome insertion (see Refs.^6,7^). The pattern of gene expression in such stable transgenic lines needs to be compared to that of the endogenous gene, as artifacts in the pattern of gene expression can be caused by the incomplete promoter fragments used and presence of vector sequences,^8,9^ or by positional effects of transgene insertion into the genome.^8,10^

**FIG. 1.**
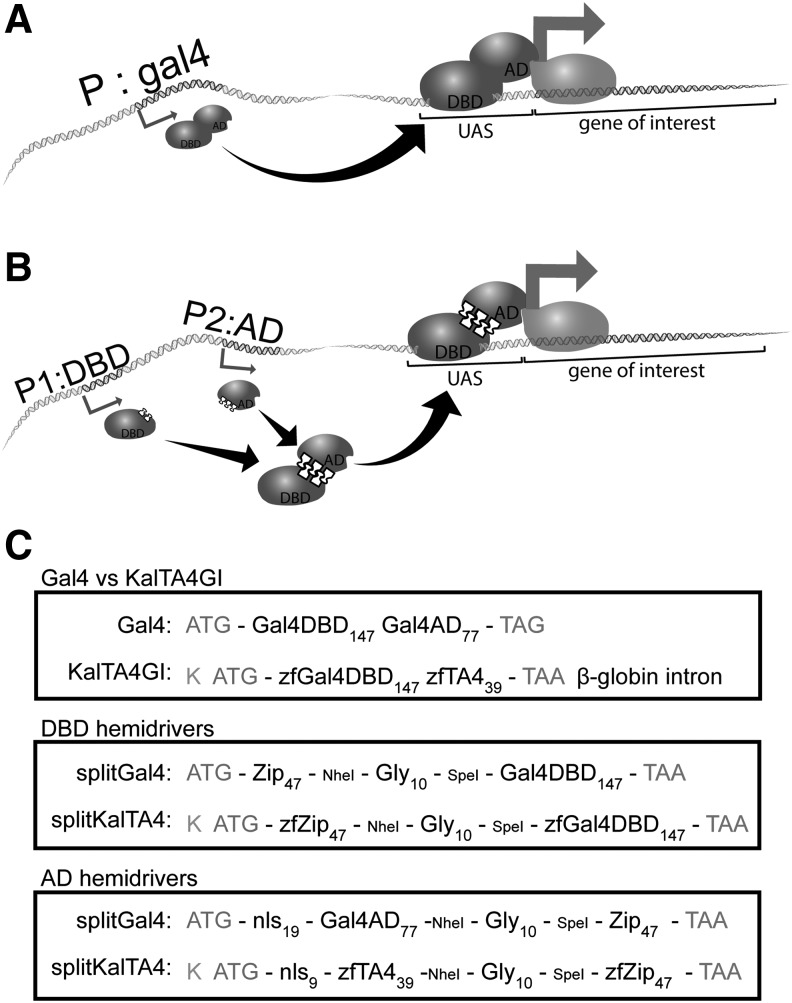
Overview of Gal4, Split Gal4, KalTA4, and Split KalTA4 systems. **(A)** Gal4/UAS system: Gal4 driver lines contain a transgene encoding Gal4 (DBD and AD in a single open-reading frame) under a specific promoter (P). In a cell where P is active, Gal4 protein is expressed and can bind UAS in effector lines, activating transcription of the downstream gene-of-interest (*gray arrow*). **(B)** Split Gal4 system: Gal4 DBD hemi-driver is encoded under one promoter (P1) and Gal4 AD hemi-driver in a separate open-reading frame under a different promoter (P2). In a cell where both promoters are active, the two domains assemble and reconstitute Gal4 function, leading to expression of genes-of-interest only in the intersection of two expression domains. **(C)** Structure of Gal4, KalTA4, split Gal4, and split KalTA4 coding sequences. Compared to Gal4, KalTA4 includes a stronger Kozak sequence (K), zebrafish-optimized codon usage (zf), the core repeats of the VP16 AD (TA4), and a β-globin intron; these features were used to generate the split KalTA4 hemi-drivers. ATG: start codon, TAG/TAA: stop codon, Gly: decaglycine linker, Zip: heterodimerizing leucine zippers, nls: nuclear localization signal. *Numbers* indicate length of feature in amino acid residues. NheI and SpeI restriction sites from the original split Gal4 constructs were maintained in split KalTA4. AD, activation domain; DBD, DNA-binding domain; UAS, upstream activating sequence.

Researchers can also target gene expression by resorting to the bipartite Gal4/UAS system, amply used in Drosophila^3,11^ and in zebrafish^12–15^ to drive gene expression in particular cell types (Fig. 1A). A Gal4 driver line expresses the yeast transcription factor Gal4 in a specific pattern (for instance, under the control of a previously characterized promoter fragment). Driver lines are crossed with effector lines, where an upstream activating sequence (UAS), specifically recognized by Gal4, is cloned upstream of GOI. Directed expression in the target cells occurs in the double transgenic offspring. An advantage of this versatile system is that a variety of Gal4 driver lines already exist (see Refs.^13,16–18^ for examples of collections of enhancer-trap Gal4 lines), which allows researchers to swiftly target gene expression to different tissues or developmental stages by combining a UAS effector line with existing driver lines.

A striking limitation of both approaches is that gene expression is ultimately under the control of a single promoter, which may not achieve the required cell type specificity. In fact, existing Drosophila or zebrafish Gal4 driver lines, whether enhancer-trapped or genetically engineered to drive Gal4 expression by a known promoter, rarely confine expression to a single cell type.^19,20^ This may preclude the analysis of the cell autonomy of the effects of gene expression, for instance, or may not enable visualization of a particular cell type with the necessary specificity, if related or nearby cell types also express a reporter protein.

To overcome this limitation, ternary systems such as the Split Gal4 system have been developed (Fig. 1B).^21,22^ This system takes advantage of the modularity of Gal4 domains, whereby the DNA-binding domain (DBD) can be encoded separately from the activation domain (AD) in so called hemi-drivers. Each domain is not functional on its own, but when coexpressed reconstitute Gal4 function and activate expression of UAS-driven transgenes. When each hemi-driver is encoded under the control of a different promoter, only those cells in which both promoters are simultaneously active synthesize both hemi-drivers. Each hemi-driver contains a heterodimerizing leucine zipper that ensures that where coexpression happens, the two hemi-drivers dimerize and reconstitute a functional Gal4, which can bind UAS and initiate transcription.^21^ This allows gene expression in the intersection of two expression patterns, when a single promoter does not provide enough specificity, allowing dissection of complex expression patterns by iterative combinations with more specific promoters. The split Gal4 system has been recently used to refine gene expression to specific neuron subsets and correlate them to a particular function or behavior (see examples in Drosophila studies here^23–29^).

In this study, we describe an adaptation of the Split Gal4 system for use in zebrafish, which we term Split KalTA4, since we based it on the optimized version of Gal4, KalTA4.^30^ Compared to the original Gal4 constructs, KalTA4 shows robust zebrafish expression due to a number of optimal modifications: (1) it includes a strong Kozak sequence and a rabbit β-globin intronic sequence that increase expression levels; (2) codon usage is optimized throughout for translation in zebrafish; (3) the entire VP16 AD is replaced with attenuated repeats of the VP16 core sequence, TA4, which is similarly potent in transgene activation, but less toxic. We design KalTA4-based hemi-drivers and confirm that they work in the developing zebrafish, and we provide an example where the use of split KalTA4 allowed the dissection of a complex expression pattern by restricting reporter expression to a subset of cells in that pattern.

## Materials and Methods

### Fish husbandry

The following wild-type and transgenic zebrafish lines were used: AB; Tg(sox10(7.2):mRFP)^31^; Tg(olig2:EGFP)^32^; Tg(mbp:EGFP)^33^; and Tg(UAS:kaede).^18^ For this study, we generated the transgenic line Tg(sox10(7.2):KalTA4GI). Throughout the text and figures, we refer to Tg(sox10(7.2):mRFP) as Tg(sox10:mRFP) and Tg(sox10(7.2):KalTA4GI) as Tg(sox10:KalTA4) for simplicity. All animals were maintained in accordance with the United Kingdom Home Office guidelines.

### Generation of DBD hemi-driver

The coding sequence for the original DBD hemi-driver^21^ encodes a heterodimerizing leucine zipper (Zip), a decaglycine linker (gly), and the DBD of Gal4 (Gal4DBD, residues 1–147), as depicted in Figure 1C. Residues 1–147 are identical in Gal4 and KalTA4, but the coding sequence has been optimized for zebrafish expression in KalTA4.^30^ We polymerase chain reaction (PCR)-amplified KalTA4's DBD coding sequence from pCSKalTA4^30^ using Phusion High-Fidelity DNA Polymerase (New England Biolabs) and added the remaining features by consecutive extension PCRs, using the primers described in Table 1. Following each round, we purified the PCR product of the expected size using the QIAquick Gel Extraction Kit (Qiagen) and used it as template in the subsequent round. In Table 1, italics denote KalTA4's DBD coding sequence, bold denotes the Zip sequence, and underlined is the gly sequence. Round 3 adds a start codon and strong Kozak sequence from KalTA4. In the last round of PCR, we added attB1 and attB2R sequences (lower case), and used BP clonase to recombine it with pDONR221 to generate a Gateway middle-entry vector, pME-DBD.

**Table 1.** Primers Used for Generating DBD Hemi-Driver by Extension PCRs

*Round*	*Primers (5′-3′)*
1	F: **GCTACGGCCCCCTGGGCGGCGGCAAG**GCTAGCGGAGGAGGCGGCGGAGGCGGAGGCGGAGGCACTAGT*ATGAAACTGCTCTCATCCATCG*
	R: ggggaccactttgtacaagaaagctgggtTTA*CGAAACAGTCAGCTGTCTCTG*
2	F: **CCGAGGTGGCCGAGCTGGAGCAGGAGGTGCAGCGCCTGGAGAACGAGGTGAGCCAGTACGAGACCCGCTACGGCCCCCTGGGCGGCGGC**
	R: same as Round 1
3	F: GCCGCCACCATG**CTGGAGATCCGCGCCGCCTTCCTGCGCCAGCGCAACACCGCCCTGCGCACCGAGGTGGCCGAGCTGGAGCAGG**
	R: same as Round 1
4	F: ggggacaagtttgtacaaaaaagcaggctGCCGCCACCATGCTGGAGATCCG
	R: same as Round 1

*Italics* denote KalTA4's DBD coding sequence, *bold* denotes the ‘Zip’ sequence, and *underlined* is the ‘gly’ sequence.

DBD, DNA-binding domain; PCR, polymerase chain reaction.

### Generation of TA4 hemi-driver

The coding sequence for the original Gal4 AD or VP16 hemi-drivers^21^ encodes a long SV40 nuclear localization signal (“nls”); the transcription-AD of Gal4 or VP16 (AD); a decaglycine linker (gly); and a leucine zipper complementary to that in the DBD hemi-driver (Zip), as depicted in Figure 1C. In KalTA4, which has been optimized for zebrafish,^30^ the AD consists only of the core repeats of the VP16 AD, TA4. We PCR-amplified the sequence encoding TA4 (residues 154–192) from pCSKalTA4^30^ using Phusion High-Fidelity DNA Polymerase and added the remaining features by consecutive extension PCRs, using the primers described in Table 2. Following each round, we gel-purified the PCR product of the expected size and used it as a template in the subsequent round. In Table 2, italics denote the TA4 coding sequence, bold denotes the Zip sequence, and underlined is the gly sequence. Round 1 adds a short SV40 nuclear localization signal (delimited by dashes). Round 2 adds a start codon and strong Kozak sequence from KalTA4. In the last round of PCR we added attB1 and attB2R sequences (lowercase) and used BP clonase to recombine it with pDONR221 to generate a Gateway middle-entry vector, pME-TA4.

**Table 2.** Primers Used for Generating TA4 Hemi-Driver by Extension PCRs

*Round*	*Primers (5′-3′)*
1	F: CCACCATGGATAAA-ATGGCTCCAAAGAAGAAGCGTAAGGTA-*CGGCCGACGCCCTGGACG*
	R: **CCAGGAAGGCGGCCTCGATCTCCAG**ACTAGTGCCTCCGCCTCCGCCTCCGCCGCCTCCTCCGCTAGC*GAGGATGTCCAGGTCGTAGTCG*
2	F: ggggacaagtttgtacaaaaaagcaggctGCCGCCACCATGGATAAA
	R: **GCGCAGGCGCTGCACGCGCTGGCGCAGCTCGGCCACGCGGGTCTCCAGGGCGGTGTTCTCGCGCTCCAGGAAGGCGGCCTCGATCTCC**
3	F: same as Round 2
	R: TTA**CTTGCCGCCGCCCAGGGGGCCGTAGCGGGTGCGGTTCTGGCTCACGCGGTTGCGCAGGCGCTGCACGCGCTGG**
4	F: same as Round 2
	R: ggggaccactttgtacaagaaagctgggtTTA**CTTGCCGCCGCCCAGGGGGC**

*Italics* denote the TA4 coding sequence, *bold* denotes the ‘Zip’ sequence, and *underlined* is the ‘gly’ sequence.

During primer design, codon usage was optimized for zebrafish expression by using the most frequently used codon for each particular residue according to Yarden.^34^ Constructs were verified by Sanger sequencing (Source BioScience LifeSciences), and are available upon request, as are their sequences.

### Generation of DBD and TA4 synthetic mRNAs

The Kozak and coding sequences for the DBD and TA4 hemi-drivers were PCR-amplified using Phusion High-Fidelity DNA Polymerase and cloned into pCS2+ digested with BamHI and Klenow blunted. Constructs were verified by Sanger sequencing. Synthetic mRNA was produced by linearizing the templates with *Not*I and transcribing from the SP6 promoter using SP6 mMESSAGE mMACHINE (Ambion).

### Gateway recombination of split KalTA4 hemi-drivers

We used LR clonase II Plus (Life Technologies) to recombine 5′-entry vectors containing a fragment of the h2α (Kwan *et al.*^7^), *sox10* (Kirby *et al.*^31^), or *elavl3* (formerly huC) (Park *et al.*^42^) promoters; middle-entry vectors pME-DBD, pME-TA4, or pME-KalTA4GI; a 3′-entry vector containing a poly adenylation signal from the Tol2kit^7^ and destination vectors pDestTol2pA2 or pDestTol2CG2 (for sox10(7.2):KalTA4GI line) to generate the constructs used in this study. All entry vectors were verified by Sanger sequencing and all recombined constructs were screened by at least three restriction enzyme digestions.

### Microinjection

For DNA injections, 2–10 pg of DNA of each construct with 30–50 pg of tol2 transposase mRNA were microinjected in a volume of 1 nL into the cell(s) of fertilized UAS:kaede eggs (1–4 cells stage). For synthetic mRNA injections, 100 pg of each mRNA in water was microinjected in a volume of 1 nL into the yolk of fertilized Tg(UAS:kaede) eggs (eggs injected with both hemi-drivers contained a total amount of 200 pg mRNA). The injection solution contained 0.05% Phenol red for visualization.

For the generation of the stable transgenic line Tg(sox10(7.2):KalTA4GI), plasmid DNA encoding sox10:KalTA4 and tol2 transposase mRNA were injected as described in one-cell stage AB fertilized eggs. Successful integration of sox10:KalTA4 into the genome was detected by cmcl2:EGFP expression in the heart. GFP+ potential founders were individually outcrossed to AB wild-type, and screened for green hearts in the offspring. A founder with germ-line transmission of sox10:KalTA4 was identified and the F1 generation was raised and also screened for stable sox10:KalTA4 genome integration.

### Live imaging

The mRNA-injected embryos were screened for Kaede fluorescence from 24 hpf in a Nikon SMZ1500 stereomicroscope equipped with a Nikon Intensilight Illuminator. Plasmid-injected embryos/larvae were embedded in 1.5% low melting point agarose in 10 mM HEPES-buffered E3 embryo medium with tricaine (168 μg/mL 3-amino-benzoic acid ethyl ester) and screened for Kaede+ fluorescence using a Zeiss Axio Imager A1 microscope equipped with a 20× objective. A check for Kaede photoconversion from green to red fluorescence was performed using UV illumination for 30–60 s. Select embryos/larvae were live-imaged using either a Zeiss Axio Imager Z1 equipped with an Apotome.2 unit and a 10× objective, or a Zeiss LSM710 confocal microscope equipped with a 20× objective, or a Zeiss 780 confocal microscope equipped with a 63× water immersion objective. All fluorescent spinal cord images represent a lateral view, anterior to the left and dorsal to the top. Image processing was kept to a minimum and included only cropping and global changes of brightness and contrast, and was performed in Fiji software (a distribution of ImageJ). Figure panels were produced using Adobe Illustrator CS8.

### Fluorescence measurements

Fluorescence intensity measurements were performed in Fiji. Briefly, two to four optical sections of the z-stack encompassing the soma of randomly selected muscle or neuronal cells were z-projected. A threshold was applied to select the soma of the cells, and the measure tool was used to determine the Mean Gray Value, reflecting the average fluorescence intensity across the entire soma area.

## Results

### Design of split KalTA4 hemi-drivers

To generate a conditional expression system in zebrafish where GOI are expressed in the intersection of expression patterns, we adapted the split Gal4 system developed for Drosophila,^21^ where the DBD and the AD of the transcription factor Gal4 are encoded in separate hemi-drivers. To facilitate assembly of a functional transcription factor when hemi-drivers are coexpressed, Luan *et al.* fused a heterodimerizing leucine zipper to each hemi-driver (Fig. 1B, C).^21^ We designed hemi-drivers based on the original sequences, splitting instead KalTA4, a version of Gal4 optimized for zebrafish (see Fig. 1C for construct design).^30^

We used the sequence encoding residues 1–147 of KalTA4 as a starting point for the DBD hemi-driver, and residues 154–192 as a starting point for the TA4 hemi-driver, and added the remaining features by extension PCR (see Materials and Methods section for details). In addition to the decaglycine linker and the heterodimerizing leucine zippers, we changed the original nuclear localization signal (nls) sequence of the AD hemi-driver to the shorter SV40 nls sequence. Similar to the KalTA4 constructs, we also included a strong Kozak sequence in both hemi-drivers, and also optimized codon usage in all features for expression in zebrafish. In our constructs, only the plasmids encoding intact KalTA4 (positive control) included the rabbit β-globin intron downstream of the coding sequence, which is a modification reported to increase the expression originally included in the design of KalTA4.^30^ All constructs were verified by Sanger sequencing.

### Expression of both hemi-drivers is required to transactivate a reporter transgene *in vivo*

**FIG. 2.**
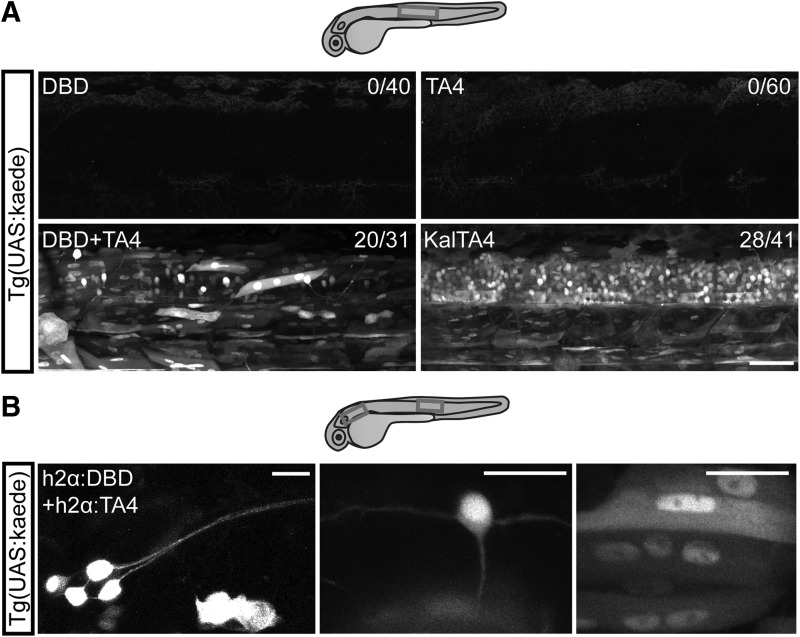
Split KalTA4 hemi-drivers reconstitute functional KalTA4 in several cell types. **(A)** Lateral view of the trunk region of 2 dpf Tg(UAS:kaede) larvae injected with split KalTA4 hemi-driver mRNA or control KalTA4 mRNA. Either DBD or TA4 mRNA alone does not activate kaede expression. DBD and TA4 mRNA in combination, or intact KalTA4 mRNA, is sufficient to activate the reporter UAS:kaede. Since heterozygous parent UAS:kaede fish were outcrossed, the subset of nonfluorescent embryos are likely nontransgenic offspring. *Numbers* on *top right* indicate number of kaede+ embryos/number of total embryos. *Boxes* indicate approximate imaged regions. Scale bar: 50 μm. **(B)** Lateral view of the pLL ganglion (*left*) or trunk region (*middle* and *right*) of 2 dpf Tg(UAS:kaede) larvae injected with h2α:DBD and h2α:TA4. h2α-driven split KalTA4 expression results in activation of the reporter UAS:kaede in a variety of cell types, including peripheral neurons in the pLL ganglion (*left*), central neurons in the spinal cord such as a commissural primary ascending interneuron (*middle*) and muscle cells (*right*). *Boxes* indicate areas of imaged cells. Scale bars: 20 μm. pLL, posterior lateral line.

We first determined whether the modifications we inserted into the system worked *in vivo*. We sought to ubiquitously express both hemi-drivers in zebrafish embryos. We injected synthetic mRNA of each hemi-driver in isolation or combined, and of intact KalTA4, into fertilized Tg(UAS:kaede) eggs. mRNA injection results in ubiquitous expression in the early embryo, and if the split KalTA4 hemi-driver proteins assemble into a functional KalTA4, it will bind the UAS sequence and drive expression of the fluorescent protein Kaede in the embryo. When mRNA for each hemi-driver was injected alone, expression of Kaede was not detected at any stage analyzed (up to 4 dpf; Fig. 2A; DBD: 0/40 embryos; TA4: 0/60 embryos). When mRNAs for both hemi-drivers were coinjected, clear expression of Kaede was detected from at least 24 hpf (Fig. 2A, at 48 hpf, 20/31 embryos). Injection of intact KalTA4 mRNA similarly results in the activation of Kaede expression throughout the embryo (Fig. 2A, 28/41 embryos). Thus, our modifications to the split Gal4 system, which we term split KalTA4, are functional *in vivo*, and recognition of the UAS sequence and activation of downstream gene transcription requires the presence of both Split KalTA4 hemi-drivers.

### Split KalTA4 functions in a variety of cell types and with similar efficiency to intact KalTA4

We then generated DNA constructs where each hemi-driver's coding sequence is preceded by a fragment of the histone 2-α promoter, which drives the expression in many cell types (Kwan *et al.*^7^). Similarly to the mRNA microinjections, only coinjection of h2α:DBD with h2α:TA4 or injection of the intact h2α:KalTA4 resulted in activation of the UAS:kaede reporter (h2α:DBD: 0/20 embryos; h2α:TA4: 0/20; h2α:DBD and h2α:TA4: 29/35 embryos; h2α:KalTA4: 23/45 embryos). The use of the quasi-ubiquitous h2α promoter and of DNA constructs resulted in sparser expression than mRNA injections and allowed us to identify by morphology and location the cell types expressing the reporter fluorescent protein Kaede. Similar to intact KalTA4, coinjections of the split KalTA4 hemi-drivers resulted in Kaede expression in both central and peripheral neurons, muscle cells (Fig. 2B), skin cells, and notochord cells. Thus, the hemi-drivers can heterodimerize and transactivate the reporter transgene in many cell types.

We then wondered if reconstituted split KalTA4 activated the reporter transgene with the same efficacy as intact KalTA4. We considered fluorescence intensity as a proxy for the level of UAS:kaede expression and transactivation efficiency. We measured the average fluorescence intensity of the soma area of randomly selected Kaede+ neurons or muscle cells. Intensity in Kaede+ muscle cells driven by split KalTA4 was similar to intensity in Kaede+ muscle cells driven by intact KalTA4 (34 ± 23 AU in 24 h2α:DBD + h2α:TA4 cells versus 31 ± 15 AU in 21 h2α:KalTA4 cells, *p* = 0.8482 in Mann–Whitney test). The intensity in Kaede+ neurons driven by split KalTA4 was slightly reduced compared to the intensity in Kaede+ neurons driven by intact KalTA4 (84 ± 42 AU in 20 h2α:DBD + h2α:TA4 neurons versus 109 ± 27 AU in 20 h2α:KalTA4 neurons, *p* = 0.0122 in Mann–Whitney test). This suggests that the efficiency of Split KalTA4 transactivation of the transgene may be slightly variable between cell types, but can readily reach similar levels to intact KalTA4 *in vivo*.

### Split KalTA4 restricts sox10 expression pattern to smaller subsets *in vivo*

One potential application of split Gal4/KalTA4 systems is to dissect complex gene expression patterns to its constituents, driving GOI expression in increasingly specific subsets of cells. The *sox10* gene encodes an important transcription factor that regulates the development of many cells at several stages, including some neuronal subtypes, neural crest, its derivatives, and myelinating glia.^35–38^ Fragments of the promoter of the zebrafish *sox10* gene drive the expression of reporter genes in several of these cell types.^39^ For instance, in the Tg(sox10:mRFP) line,^31^ a 7.2 kbp fragment of the *sox10* promoter drives the expression of mRFP in central neurons, glia, and neural crest-derived cells.^40^

**FIG. 3.**
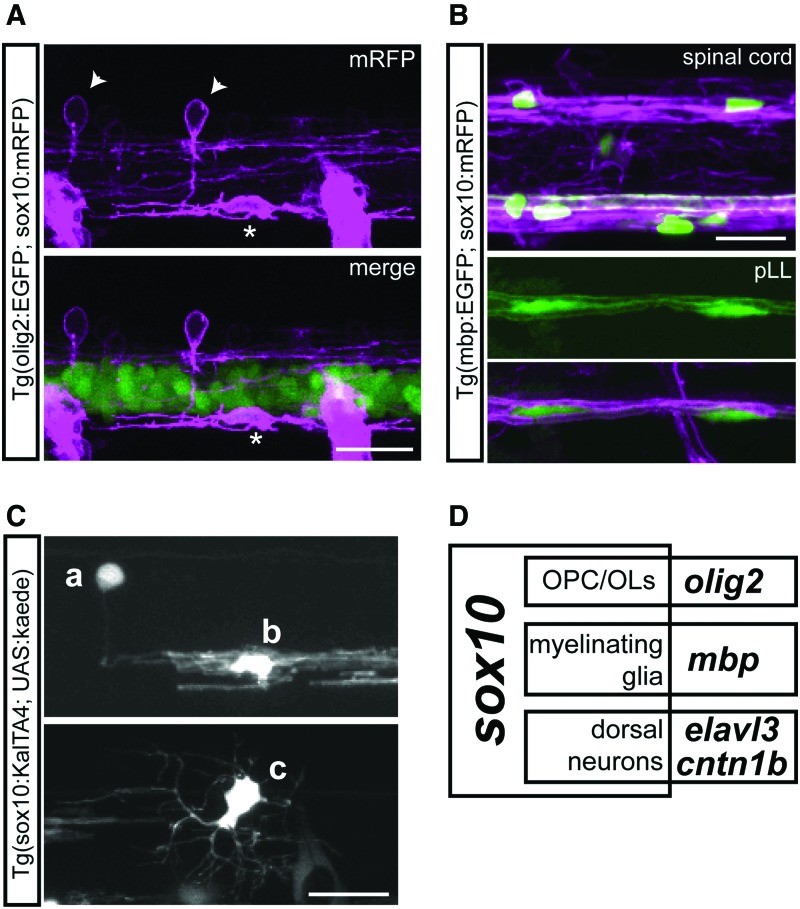
Reporter transgenes indicate expression of *sox10* in several cell types. **(A)** Lateral view of the spinal cord of 2 dpf Tg(olig2:EGFP; sox10:mRFP) larva. *Arrowheads*: examples of sox10:mRFP+ dorsal neurons; *asterisk*: example of olig2:GFP+ sox10:mRFP+ OPC. **(B)** Lateral view of the spinal cord (*top*) and pLL (*bottom*) of 4 dpf Tg(mbp:EGFP; sox10:mRFP) larva. Many OPCs become mbp:EGFP+ mature OLs by 4 dpf in the spinal cord, and are associated with sox10:mRFP myelin sheaths, as are mbp:EGFP+ Schwann cells in the pLL. **(C)** Lateral view of the spinal cord of 3 dpf Tg(sox10:KalTA4; UAS:kaede) larva. Cell a: dorsal neuron; cell b: OL; cell c: OPC. **(D)** Venn Diagram of the expression patterns of *sox10*, *olig2*, *mbp*, *elavl3*, and *cntn1b*, indicating some of the cell types found in the intersection of each subdomain. All scale bars: 20 μm. OL, oligodendrocyte; OPC, oligodendrocyte precursor cell. Color images available online at www.liebertpub.com/zeb

At 2 dpf, we find that a subpopulation of sox10:mRFP+ dorsal neurons is evident in the embryonic spinal cord (Fig. 3A, arrowheads), in addition to oligodendrocyte precursor cells (OPCs). Unlike the dorsal neurons, OPCs also express the transcription factor Olig2, and are labeled by EGFP in the Tg(olig2:EGFP) line (Fig. 3A, asterisk). Later, at 4 dpf, fewer dorsal neurons are apparent, suggesting downregulation of *sox10* expression in these neurons. Many OPCs have terminally differentiated into myelinating oligodendrocytes (OLs), which retain expression of *sox10* and initiate expression of myelin basic protein (*mbp*), becoming labeled in the Tg(mbp:EGFP) line (Fig. 3B, spinal cord). In the peripheral nervous system, myelinating Schwann cells in the posterior lateral line (pLL) also express *sox10* and *mbp* (Fig. 3B, pLL).

To exclude the possibility that such an expression pattern is specific to this Tg(sox10:mRFP) line (for instance, due to positional effects of original transgene insertion), we generated a different stable transgenic line using the same *sox10* promoter fragment, driving intact KalTA4, Tg(sox10:KalTA4). When crossed with the reporter Tg(UAS:kaede), *sox10*+ cells are sparsely labeled in the offspring, permitting visualization of their morphology and unambiguous identification. Such sparse, variegated labeling in stable reporter UAS transgenic lines is thought to be due to epigenetic silencing of the repetitive UAS sequences,^41^ but in this case is useful to identify isolated cells and more completely visualize their individual morphology. Similar to the Tg(sox10:mRFP) line, analysis of the Tg(sox10:KalTA4) line at 3 and 4 dpf confirmed that within the spinal cord, *sox10* is expressed in some dorsal neurons (Fig. 3C, cell a), mature OLs (Fig. 3C, cell b), and OPCs (Fig. 3C, cell c).

We then used the split KalTA4 system to restrict expression of the reporter transgene to subtypes of *sox10*+ cells, in transient expression assays. Most newly differentiated neurons also express *elavl3*, which encodes an RNA-binding protein used as a pan-neuronal marker.^42^ Thus, we sought to label cells only in the intersection of the *sox10* and *elavl3* expression patterns (Fig. 3D); we generated hemi-driver constructs preceded by fragments of the *sox10* and *elavl3* promoters and injected different combinations of the hemi-drivers in the reporter UAS:kaede line. As is typical of injected DNA constructs, these transient expression assays resulted in sparse, mosaic Kaede expression. We identified Kaede+ cells in multiple injected embryos for each combination of constructs, to establish a consensus expression pattern for each combination. Table 3 shows the number of injected larvae screened at 4 dpf with Kaede+ cells of several cell types.

**Table 3.** Summary Table: Number of Injected Animals with Kaede+ Cells of Each Cell Type

		*Neurons*							*Glia*				
*DBD*	*TA4*	*all*	*pLL*	*RB*	*B*	*RS*	*IN*	*Mn*	*OL*	*SC*	*M, N, FP*	*Zero cells*	*No. of embryos*
*elavl3*	—	0	0	0	0	0	0	0	0	0	0	51	51
—	*elavl3*	0	0	0	0	0	0	0	0	0	0	24	24
*elavl3*	*elavl3*	4	1	10	10	2	14	5	0	0	10	54	69
*sox10*	—	0	0	0	0	0	0	0	0	0	0	35	35
—	*sox10*	0	0	0	0	0	0	0	0	0	0	27	27
*sox10*	*sox10*	1	2	3	14	4	16	4	4	8	23	28	51
*sox10*	*elavl3*	6	8	5	18	3	23	9	0	0	25	34	60
*elavl3*	*sox10*	7	7	3	27	13	31	18	0	0	30	28	60
*sox10*	*cntn1b*	1	0	0	3	2	10	0	0	0	24	31	60

all, anterior lateral line; B, brain neurons; FP, floorplate cells; IN, spinal interneurons; M, muscle; Mn, motoneurons; N, notochord; OL, oligodendrocytes; pLL, posterior lateral line; RB, Rohon-Beard neurons; RS, reticulospinal neurons; SC, Schwann cells.

First, injection of each hemi-driver alone, preceded by either the *elavl3* promoter fragment or the *sox10* promoter fragment, did not yield any fluorescent reporter cells, confirming the specificity of our system, as also determined by our previous injections (Fig. 2).

**FIG. 4.**
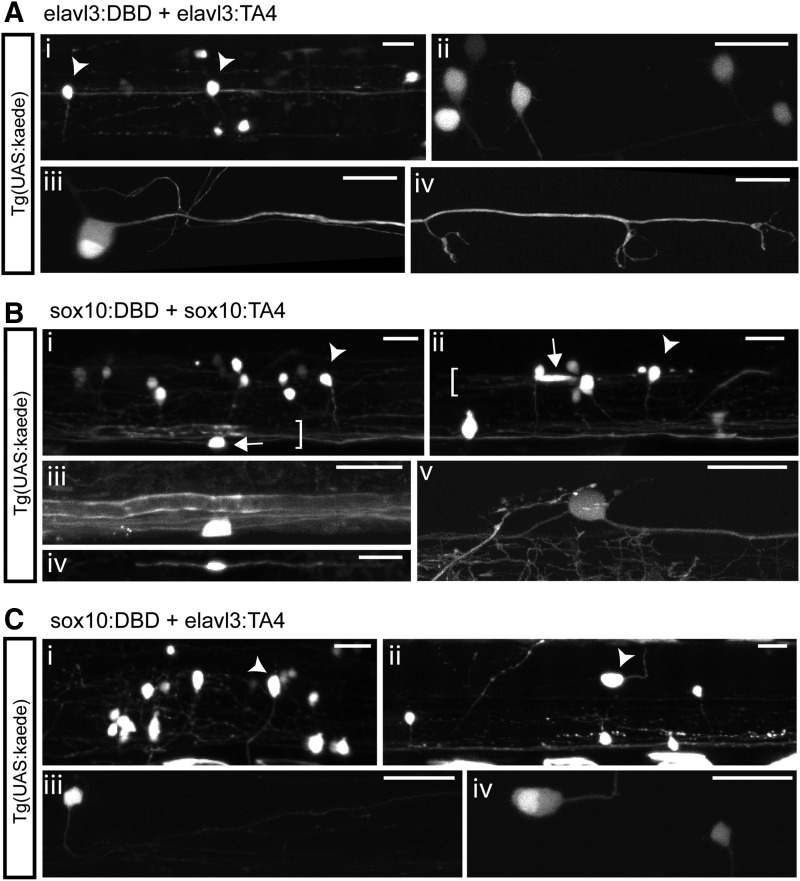
Split KalTA4 system restricts *sox10* expression pattern to each component cell type. **(A)** Coinjection of elavl3:DBD and elavl3:TA4 activate reporter expression in neurons. **(i)** Overview of two spinal cord segments showing kaede+ neurons (*arrowheads* indicate examples of neuron somas). **(ii)** High-resolution view of spinal interneurons. **(iii–iv)** A pLL neuron and its axon innervating neuromasts. **(B)** Coinjection of sox10:DBD and sox10:TA4 activate reporter expression in neurons and glia. **(i, ii)** Spinal cord overviews showing kaede+ neurons (*arrowheads*) and OLs (*arrows*) forming myelin sheaths (*brackets*). **(iii)** High-resolution view of ventral OL in **(i). (iv)** A Schwann cell in the pLL. **(v)** High-resolution view of a Rohon-Beard cell in the dorsal spinal cord. **(C)** Coinjection of sox10:DBD and elavl3:TA4 activate reporter expression only in neurons. **(i, ii)** Spinal cord overviews showing kaede+ neurons (*arrowheads*). **(iii)** High-resolution view of a circumferential descending interneuron. **(iv)** High-resolution view of Rohon-Beard neuron and of a dorsal interneuron in **(ii)**. All panels are lateral views at 4 dpf; all scale bars: 20 μm.

Second, coinjection of hemi-drivers preceded by the same promoter fragment restored expression typical of that promoter fragment. Injection of elavl3:DBD with elavl3:TA4 (see example in Fig. 4A) yielded expression only in neuronal cells (in 15/69 injected Tg(UAS:kaede) larvae, 22%—note that only 50% of the total number of injected larvae would carry the transgene, as heterozygous UAS:kaede parents were crossed to wild-type fish). Table 3 shows a breakdown of identified specific neuronal subtypes, and these included central and peripheral neurons. No glial cells were identified. In contrast, injection of sox10:DBD and sox10:TA4 (see example in Fig. 4B) yielded expression in neuronal cells (in 19/51 injected UAS:kaede larvae, 37%), but also in glial cells (in 9/51 larvae, 18%).

Note that, in both cases, some muscle cells, notochord, and floor plate cells were additionally labeled (M, N, FP column in Table 3). These cell types often misexpress transgenes from injected plasmid DNA in such transient assays, as has also been detected by others.^9,39^ These ectopic sites of expression were never labeled when each hemi-driver was injected separately, further highlighting that split KalTA4 hemi-drivers are not leaky.

Finally, when a hemi-driver preceded by the *elavl3* promoter was coinjected with a hemi-driver preceded by the *sox10* promoter, expression restricted only to the intersection of the two promoters' expression domains–neuronal cells. When sox10:DBD was combined with elavl3:TA4 (see example in Fig. 4C), we obtained 26/60 larvae (43%) with expression in several neuronal subtypes, but no expression in glial cells. To check whether the DBD or the TA4 hemi-driver themselves conferred any particular specificity to the system, we swapped hemi-drivers around. When elavl3:DBD was combined with sox10:TA4, we similarly observed expression only in neuronal subtypes, in 31/60 larvae (52%), and none in glial cells. All individual neuronal subtypes that were labeled in the first combination were also labeled when we swapped the hemi-drivers around.

Furthermore, we also coinjected the DBD hemi-driver preceded by the *sox10* promoter fragment with the TA4 hemi-driver preceded by a fragment of the *contactin1b* (*cntn1b*) promoter, which we have shown to be expressed in several neuronal subtypes, especially spinal neurons.^43^ In this combination of hemi-drivers, only Kaede+ neurons were found, in 14/60 larvae (23%), and no glial cells were detected. The most frequent subpopulation of neurons labeled was that of dorsal, spinal interneurons. This result indicates that the *sox10*+ subset of neurons in the dorsal spinal cord also express *contactin1b*, and confirmed that the split KalTA4 system can be used to restrict reporter expression to a cell type in the intersection of two different expression patterns.

Thus, the split KalTA4 system can be to target GOI expression to spatially restricted cell populations, dissecting a promoter fragment's complex expression pattern.

## Conclusions

We have adapted an intersectional expression system, Split Gal4, for use in zebrafish, and named it Split KalTA4. In this ternary system, two distinct promoters drive expression of a hemi-driver each, and in those subsets of cells in which both promoters are active, hemi-drivers reconstitute the transcription factor KalTA4, resulting in activation of a UAS-driven GOI in the intersection of two expression patterns. We showed that our modifications to the system function in living zebrafish. These principally include zebrafish-specific adaptations that were part of the original KalTA4 optimization.

Careful fluorescent analysis of stable transgenic *sox10* fluorescent reporters showed that in the embryonic spinal cord, a subset of dorsally located neurons also express *sox10*. We provided a specific example for the use of the split KalTA4 system, in which we restricted the broad *sox10* expression pattern to distinct components, neuronal or glial. Researchers wishing to analyze the OL lineage cell population should bear in mind that imaging solely the sox10:mRFP line at early stages will, therefore, additionally include neuronal cell profiles. The split KalTA4 system allowed us to restrict reporter expression to the neuronal population, by combining *sox10* and *elavl3* hemi-drivers. Furthermore, the combination of *sox10*- and *cntn1b*-driven hemi-drivers also restricted UAS-driven reporters solely to neurons, indicating the *sox10*+ dorsal neurons also express *contactin1b.* Thus, the split KalTA4 system may aid in finely resolving the expression pattern of promoter fragments.

The functionality of the Split KalTA4 system may be further expanded by generating hemi-drivers preceded by the heat-shock inducible promoter *hsp70*, which would additionally allow temporal control over expression of GOI. Furthermore, the hemi-drivers themselves may be further optimized: for instance, in the TA4 hemi-driver, the TA4 configuration of the core VP16 activation repeats can be replaced with configurations less efficient (TA6) or more efficient (TA2),^44^ providing an additional level of control of GOI expression. Generating hemi-drivers based on the tamoxifen-inducible Gal4-ERT protein^45^ may also allow temporal control of gene expression, while permitting the use of two specific promoters to drive expression of split KalTA4 hemi-drivers.

We urge researchers wishing to use the split KalTA4 system to establish full, stable transgenic hemi-driver lines, which should circumvent both the ectopic expression from the hemi-driver vectors in muscle and notochord cells, and the labeling mosaicism typical of transient DNA injections. Furthermore, use of UAS reporter lines with fewer nonrepetitive UAS sequences should help circumvent heritable methylation-based silencing and further reduce mosaicism.^41^ Finally, we recommend that researchers screen multiple potential founders and establish stable lines only from those founder animals showing faithful expression of the transgene.

We are happy to provide middle-entry vectors pME-DBD and pME-TA4 to researchers wishing to establish their own transgenic hemi-driver lines by modular recombination with relevant promoters to target their cell populations of interest. We hope the Split KalTA4 system will be useful to dissect complex expression patterns and refine genetic manipulations in zebrafish.
